# Audit of documentation accompanying referred maternity cases to a referral hospital in northern Ghana: a mixed-methods study

**DOI:** 10.1186/s12913-022-07760-6

**Published:** 2022-03-16

**Authors:** Edward Kwabena Ameyaw, Roberta Mensima Amoah, Carolyne Njue, Nguyen Toan Tran, Angela Dawson

**Affiliations:** 1 School of Public Health, Faculty of Health, University of Technology Sydney, Sydney, Australia; 2 Department of Public Health, School of Allied Sciences, University for Development Studies, Tamale, Northern Region, Ghana

**Keywords:** Referral, Audit, Maternal health, Records, Documentation, Northern Ghana

## Abstract

**Background:**

Effective referral of maternity cases, which cannot be managed at the primary healthcare level, with detailed referral forms is important for reducing possible delays in the provision of higher-level healthcare. This is the first study to audit documentation or referral forms that accompany referred maternity cases to a referral hospital in the northern region of Ghana.

**Materials and methods:**

This study employed an explanatory sequential mixed-method design, starting with a quantitative review of referral forms that accompanied all patients referred to four units (antenatal, antenatal emergency, labour and neonatal intensive care) of a referral hospital in northern Ghana. In-depth interviews were held with the heads of the four units afterwards. Descriptive statistics were computed for the quantitative data. The qualitative data was subjected to content analysis. Integration of the data occurred at the data interpretation/discussion level.

**Results:**

A total of 217 referral forms were analysed. Nearly half of the cases were referred from the Tamale Metropolis (46.5%) and 83.9% were referred for advanced care, whilst 8.3% were referred due to a lack of medical logistics and equipment such as oxygen and skilled personnel (6%). Completion rates of the referral forms were as follows: < 50% completion (*n* = 81; 37.3%), 50–75% completion (*n* = 112; 51.6%) above 75% completion (*n* = 24; 11.1%). Some of the handwriting were not legible and were quite difficult to read. The key informants stated that incomplete forms sometimes delay treatment. The head of the antenatal care unit at the referral hospital suggested professional development sessions as a strategy for supporting clinicians to fill the forms as expected.

**Conclusion:**

The Ghana Health Service should conduct regular audits, develop job aides and provide incentives for health professionals who accurately complete referral forms. Completing forms and digitizing health records can help ensure further efficiencies in the health information system and sustain good maternity referral documentation practices.

## Background

A functional referral system is essential for ensuring efficiency in the allocation and utilisation of resources at different levels of the health system across the continuum of care [1]. Effective communication among health professionals is required during referral, including the need for clear written communication [2]. Incomplete health records or documentation is often cited as an issue affecting the quality of referral processes [3, 4] and may affect the quality of care provided at the receiving facility [5].

A positive association has been found between the content of referral forms and the confidence health professionals have in their ability to care for patients who have been referred to them [6]. Referrals with limited information may lead to repeated and unnecessary tests and medical errors [7]. Thus, communicating patient’s information during referral is critical as it assists receiving health facilities to provide the required healthcare interventions [8].

In Ghana, referrals usually occur from lower levels of the health system such as community or primary care to the sub-district and tertiary levels that provide more specialised care. However, a referral can occur between facilities of the same level. The Ghana Health Service (GHS) has directed that all referrals be accompanied by fully completed referral forms [9]. This is essential in maternity cases considering the severity of issues that prompts maternal referrals, including haemorrhage and foetal distress [10, 11]. Nearly all lower level facilities in Ghana refer maternal complications to higher levels of healthcare [12]. The National Referral Policy and Guidelines clearly defines how an ideal referral should be executed [9]. During referral, a staff member from the sending facility calls and notifies the proposed receiving facility, fills the referral form, arranges transportation and one of them must accompany the patient. The initial call offers the opportunity for the sending health facility to notify the receiving facility about the case at hand, and also helps to know if the facility has the capacity to handle the case. In most cases, the patient is discharged directly home by the receiving facility without counter-referral to the initial health facility.

Considering that a substantial proportion of maternal deaths occur around intrapartum period [13, 14], effective referral with a detailed referral form can help reduce possible delays in healthcare provision at the receiving facility. As a result, an improved referral process can enhance Ghana’s prospects of achieving 70 maternal deaths per 100,000 live births and 12 neonatal deaths per 1000 as envisioned by the third Sustainable Development Goal [15]. According to the recent Maternal Health Survey (MHS), the maternal mortality rate in Northern Ghana of 45 deaths per 1000 women exceeds rates in other zones across the country including the Middle (37 deaths per 1000 women) and Coastal zones (38 deaths per 1000 women) [16]. There is a paucity of empirical evidence on the quality of maternal referral documentation in Ghana [17].

In Northern Ghana, few studies have examined documentation in healthcare. In a rural hospital in Northern Ghana, Allotey and Reidpath [18] found deficiencies in record management procedures at the antenatal clinic and maternity ward. However, this study is more than two decades old, the study did not account for how referral forms are completed. Awoonor-Williams, Bailey [19] also implemented interventions to enhance maternity referral in Northern Ghana. The interventions targeted means of transportation for maternity referral, use of referral slips and health provider escort during referral. Issues about completeness of referral forms that accompany maternal cases were not explored by the study. Hence, evidence on quality of maternal referral forms in the northern region of Ghana, where women have the highest risk of maternal mortality is lacking [20]. This study aims to address this knowledge gap by investigating the quality of documentation that is required for maternal referrals in northern Ghana.

## Methods

### Study design

This study employed an explanatory sequential mixed-method design data [21, 22]. Consequently, quantitative data were gathered and analysed before the qualitative data. The mixed methods design was applied to provide multiple perspectives on communication during referral [21, 22].

### Quantitative study

A review of referral forms that accompanied all maternity conditions that were referred to a referral hospital in northern Ghana was undertaken between 1 September 2019 and 31 March 2020. The data were directly entered into a pre-developed Ms. Excel template. The template included all elements listed on the recommended referral form by the Ghana Health Service. The items on a typical referral form in Ghana are shown in Table 1. The forms have sections with pre-defined choices as well as sections where healthcare providers freely write. Records from four units of the hospital were reviewed in the following order: (1) antenatal care unit, (2) antenatal care emergency, (3) labour ward, and (4) neonatal intensive care unit.

**Table 1:** Variables on the referral form to be completed during the referral

Identification of referring officer	Health facility information	Patient identification details	Patient’s clinical information
Name of referring officer	Date	Registration number (Unique identification number)	Presenting complaint(s)
Position	Name and address of referring facility	Name and address	Examination findings
Signature	Name and address of facility referred to	Sex	Investigation results
Date/Stamp	Time referred	Date of birth/Age	Diagnosis(es)
	Time of departure (if emergency)	Health insurance status	Treatment given
		Name and address of contact person	Reason for referring to the next level
		Phone number of contact person	Urgency of referral

### Qualitative study

The qualitative component constituted in-depth interviews with the heads of units where the record reviews were undertaken. The qualitative dimension provided an understanding of some issues that emerged from the record review. All interviews were conducted in January 2021 with the interview guide. Some of the questions on the guide were “Can you please tell me about how you think referral forms are filled for cases you receive?” and “How do you think clinicians could be better supported to fill in the forms?” All interviews were conducted in English and audio-recorded with the consent of the participants. All identifying information were removed to protect the anonymity of the unit heads and hospital.

### Analytical procedure

Data from the record review (quantitative) were cleaned and checked for consistency. We subsequently analysed with SPSS version 25. Descriptive statistics were computed; that is, the findings were summarised into percentages and frequencies. Results were presented in tables. Following categorisation by similar and previous studies [e.g. 8], completeness of filling items on the referral form was computed. Completeness was categorised into (1) forms with < 50% completion, (2) forms with 50–75% completion and (3) forms with > 75% completion. Each referral form had 28 items to be filled or completed. So in determining the completeness (or otherwise), 28 was divided by 100 and each item was assigned 0.28%. For a form to score above 75%, it should have at least 21 (out of 28) items completed. A content analysis of the qualitative interview data was conducted as described by Haggarty [23]. Content analysis constitutes the objective and systematic identification of key findings of texts to make inferences. Dominant themes and sub-themes were identified and collated. All methods were carried out in accordance with relevant guidelines and regulations.

### Mixed methods integration

Integration occurred at the data interpretation/discussion level [21, 24]. The results of the qualitative and quantitative studies were presented separately and then combined during the interpretation stage to elucidate the results of the quantitative study [21, 25]. This integration helped to identify where findings from both methods agreed (converged), offered complementary information on a common issue (complemented), or contradicted each other (dissonance or discrepancy existed) [26, 27].

### Ethics approval

The Navrongo Health Research Centre of the Ghana Health Service [NHRCIRB347] approved this study’s protocol. It was subsequently ratified by the Human Research Ethics Review Committee of the University of Technology Sydney, Australia [ETH19–4201]. Approval was also obtained from the authorities of the hospital, where the records were reviewed. For the interviews, informed consent was sought from each participant. Written informed consent was obtained from all participants by signing the consent form. Further, approval was sought to tape-record the interviews and also publish the findings. All consents were sought in the English Language.

## Results

### Survey

A total of 217 referral forms were analysed. Within the Northern region, cases were referred from twelve districts, while 14.3% of the total cases came from outside the region. Nearly half of the cases were referred from the Tamale Metropolis (46.5%), followed by Tolon District (11.5%). Meanwhile, only 0.9% (*n* = 2) were referred from each of the following districts: Gushegu Municipality, Nanumba North Municipality and Mion District.

### Reasons for maternal referral

According to the referral forms, at least eight out of ten cases were referred for advanced maternal or neonatal care (83.9%) whilst 8.3% were referred due to a lack of medical logistics and equipment such as oxygen and skilled personnel (6%) (see Table 2). The forms, however, did not provide details of the specific advanced care required. One woman was referred at her request (0.4%), and for three women, the reasons were not stated (1.4%). Some specific neonatal reasons for referral were found, including neonatal jaundice, neonatal sepsis and fetal distress.

**Table 2:** Reason for referral

Reason for referral	Frequency (n)	Percentage (%)
Advanced Care	182	83.9
Lack of medical logistics and equipment	18	8.3
Absence of Skilled Personnel	13	6
Not Indicated	3	1.4
Personal Request	1	0.4
Total	217	100

### Completeness in the filling of referral forms

As shown in Table 3, nearly all the forms were not fully completed (98.2%). Just over half of the forms (51.6%) had between 50 and 75% of their contents completed. For 11.1% of the total forms, at least 75% of their sections were filled. Some of the handwriting was not legible and were quite difficult to read.

**Table 3:** Completeness in the filling of referral forms

Completeness status	Frequency (n)	Percentage (%)
Status		
Completed	4	1.8
Not completed	213	98.2
Completeness of forms		
< 50%	81	37.3
50–75%	112	51.6
Above 75%	24	11.1
Total	217	100

Across districts, it was evident that referrals from the Kumbungu District had a greater proportion of forms with at least 75% completion (21.4%) followed by forms emanating from the Sagnarigu Municipality (18.2%) as shown in Table 4. For seven districts, no forms had more than a 75% completion rate. All forms from the Kpandi district were < 50% completed whilst half the forms from the Gushegu Municipality and the Karagah District were completed (50% each).

**Table 4:** Completeness in the filling of referral forms by district of origin

	Completion Rate		
District	< 50%	50–75%	> 75%
Tamale Metropolis	38.6	48.5	13.8
Tolon District	44.0	52.0	4.0
Outside Northern Region	32.3	58.1	9.7
Savelugu Municipality	20.0	66.7	13.3
Kumbungu District	42.9	35.7	21.4
Sagnarigu Municipality	36.4	45.4	18.2
Nanumba North District	33.3	66.7	0.0
Kpandai District	100	0.0	0.0
Yendi Municipality	0.0	100.0	0.0
Karagah District	50.0	50.0	0.0
Gushegu Municipality	50.0	50.0	0.0
Nanumba North Municipality	33.3	66.7	0.0
Mion District	33.3	66.7	0.0
Total	37.3	51.6	11.1

### Key informant interviews

Four senior staff from four units of the referral hospital participated in interviews. The units are the Labour Unit, antenatal care (ANC) Unit, ANC Emergency Unit and Neonatal Intensive Care Unit (NICU). The four participants were females, aged between 29 and 50 with qualifications at the bachelor degree level. Three were married, two were Christians, and two were Muslim. Informants were from the Ga, Dagomba, Mamprusi and Gonja ethnicities.

With respect to the completeness of filling the forms, the participants admitted that while some forms were filled out correctly, there were issues with the incomplete filling of forms that affected clinicians’ ability to deliver care.

*Some of the referral forms are well tabulated but the answers [they provide] don’t go with what is being demanded. You understand. Some of them even send the referral [form] without date. Some of them will send without the name of the woman. Some no gravidity. Some [of the forms too] the reason! Then usually they don’t write the number of hours the patient has even stayed with them. If they have given [a therapeutic intervention], they find it very difficult to write that ‘I gave this at this time’, so that if you want to continue, you will know the time that you can continue. Like when you five IV [Intravenous infusion], antibiotics TDS [ter die sumendum]. If you give me time, when the patient reaches here, I know that the next dose is due or at this time. Sometimes, it is not stated so we are always infuriated with their referrals.* (Head, Labour Unit)

Another participant estimated that only “5 out of 10” of referral forms were completed correctly.

One participant felt that junior staff were not completing forms correctly.

*The referral forms are mainly for doctors so they are to fill them. If the right officer, like a senior rank nurse, midwife, a medical officer or Physician Assistant fills, that will be fine. Those people when they fill hardly make mistakes with the forms. But the mistake usually comes from the junior ranks, [and] maybe the person is not familiar with the job. That is the problem.* (Experienced Staff, NICU)

It was reported that some patients present without referral forms.

*Some come with referral forms but others don’t. They just come and tell you they are not well, but when you tell them to go back and then get a referral letter and return, they are not willing to go. So sometimes it is difficult, due to the state or the condition of the persons, [it is difficult] to even ask the person to go back and get the referral and come back.* (Head, ANC Unit)

The key informants stated that incomplete forms could delay treatment and some clinicians asked women to return to the previous facility to obtain a form. Other clinicians were reported to have taken additional time to assess the woman to determine the issue. *In those cases [problematic referral forms] we have to ask the client, probe further to get the real problem that she is presenting.”* (Head, ANC Unit). In other cases where there is a lack of information, phone calls are made:

*We do a follow up call. Sometimes they come with an ambulance or an accompanied nurse or if we have the referral facility’s number we call. Some of them add their numbers, like the officer referring. So we call those numbers.* (Experienced Staff, NICU)

One participant noted that, referral forms from the larger health facilities were usually accurate “*But hardly do we get mistakes from the big or reputable hospitals.*” (Experienced Staff, NICU).

The health professionals shared their views on how best clinicians at the referral facilities can be supported to fill the forms as expected. The head of the ANC unit suggested professional development sessions for staff stated:

*A workshop should be held and they should be taught how to fill the forms. At the CHPS compound, most of them are community health nurses and then midwives. They should be taught how to fill the form and fill it appropriately; sign it, write their names, their rank and then the phone number should be on it.* (Head, ANC Emergency Unit)

## Discussion

This mixed method study involved an audit of documentation accompanying maternity cases referred to a referral hospital in northern Ghana from September 2019 to December 2019. The qualitative dimension provided an explanation of the quantitative results. The main reasons for referral to the referral hospital included the need for advanced care and a lack of equipment such as oxygen and skilled personnel. Handwritten information on some of the forms were not legible. Only one woman requested to be referred, and the other 216 were referred on the advice of a hospital staff. Most of the referral forms were not fully completed. Health providers interviewed stated that incomplete forms affected the quality and efficiency of the care provided at the referral facility. These issues with referral documentation have been reported in other studies [28–30]. Similarly, earlier studies from Ghana have found gaps, including a lack of essential information such as the diagnosis, findings of obstetric examinations, and details concerning the care provided to manage the women’s conditions [8, 31].

Similar to the documentation gaps found in this study, a study by Osinaikea, Esezobor [32] from Nigeria, concluded that referral letters to the Lagos University Teaching Hospital were poorly written and characterised by missing information about adverse clinical conditions and the level of urgency. Only 28.4% of the referral letters had all or some information concerning the outcome of clinical investigations conducted [32]. Moreover, in Afghanistan, Kim, Tappis [33] identified issues with the completeness of records. A review indicated that several charts lacked information on the patient’s characteristics, indications, and operative or post-operative procedures. Information on partograph use was missing in 38% of the cases and information on parity was absent for 23% of the cases. Similar observation were made for information on fetal outcomes, indications for cesareans, as well as emergency categorisation [33].

The gaps in referral documentation indicate the need for concerted efforts by the Ghanaian central government and partner organisations to ensure that providers abide by the referral recommendations and guidelines issued by the Ministry of Health [9]. Regular professional development training, supportive supervision and feedback are required for health providers at lower healthcare levels to model the accurate completion of referral forms [8].

Job aides may be a useful tool to support health providers to improve the quality of referall documentation [34, 35]. The Ministry of Health could explore the use of audiovisual resources on the appropriate completion of referral documentation that could be posted around the antenatal, labour, maternity, and neonatal wards of the lower-level health facilities to serve as constant reminders on the need ensure accurate completion of referral forms.

Ensuring accurate and complete referral forms can be achieved when records keeping activities are regularly audited. In addition, health professionals who complete forms accurately could be acknowledged using financial or non financial incentives. For instance, management of health facilities can develop a citation in honour of each healthcare provider who completes referral forms as required each year. Consistent evidence indicate that intermittent record audits enhance health provider performance and care delivery [36, 37]. A qualitative study in Indonesia revealed that a lack of motivation compromises the adequate completion of health records, hence the need to motivate health care providers to ensure high quality documentation [38].

Efforts to enhance accurate and complete health records need to be undertaken alongside health information system strengthening initiatives. This includes strengthening the digitisation of records and enhancing internet connectivity to better link facilities and support communication. Illegible handwriting sometimes causes confusion and death [39]. Digitisation, in particular, can help overcome the challenge of illegibly written information and its associated consequences [40]. Internet connectivity is intermittent in most parts of the Northern region, making email communication between facilities challenging [41]. Currently, the teaching hospital manages patients’ information with a software, however, the software does not link or communicate with the lower level health facilities within the region. However, Short Message System (SMS) or mobile telephone text messages between health staff may be reliable and useful [42].

Electronic record systems enhance the efficiency of healthcare delivery, motivate healthcare providers and are essential for referral [43–46]. A study from Limpopo province in South Africa revealed that the electronic health record system saved time for recording patients’ information, enhanced communication, and facilitated confidentiality of the patients’ health records [47]. Similarly, health providers at the referred hospital did not have to re-write the patients’ clinical history [47]. Esquivel et al. [48] have advanced that for the electronic health record system to be useful in referrals, it must have some key attributes such as being reliable, timely, secure and actionable [48]. In Northern Ghana, Abdulai and Adam [49] noted that 54.9% of surveyed health professionals were ready for electronic health record keeping.

Among the core reasons for referral were the need for advanced care, the lack of essential equipment and resources, medicines and oxygen as well as the lack of skilled personnel at the time of the referral. These issues have been previously acknowledged in Northern Ghana for several decades [50]. Appropriate investment in maternal health human and material resources may reduce the need for some referrals in the first place, therefore reducing the workload at higher tier facilities.

## Strengths and limitations of the study

A major strength of the study is the mixed methods design. This design aided to elucidate the quantitative findings with the qualitative findings. Second, our search proved that no study has been conducted in Northern Ghana on referral documentation. As a result, this is the first study to investigate the filling of referral forms in the region. However, it was limited to only maternity cases; hence, other cases referred to the hospital were not audited by this study. Clinical diagnoses of the cases were omitted because the focus of the study was to investigate the depth of documentation during referral but not diagnoses.

## Conclusion

The study has indicated that frontline maternity healthcare providers in northern Ghana may require training and support for improvement in referral forms completion. Central government commitment to sustainable interventions such as in-service training and job aides are required. The Ministry of Health and the Ghana Health Service should conduct regular audits, develop job aides and provide incentives for health professionals who accurately complete referral forms. Completing forms and digitizing health records will help ensure further efficiencies in the health information system and sustain acceptable maternity referral documentation practices.

## Data Availability

All data supporting the manuscript are within the manuscript.

## Abbreviations

ANC: Antenatal Care
GHS: Ghana Health Service
MHS: Maternal Health Survey
NICU: Neonatal Intensive Care Unit
SMS: Short Message System
